# Characterization of a Pyrethroid-Degrading *Pseudomonas fulva* Strain P31 and Biochemical Degradation Pathway of D-Phenothrin

**DOI:** 10.3389/fmicb.2018.01003

**Published:** 2018-05-16

**Authors:** Jingjing Yang, Yanmei Feng, Hui Zhan, Jie Liu, Fang Yang, Kaiyang Zhang, Lianhui Zhang, Shaohua Chen

**Affiliations:** ^1^State Key Laboratory for Conservation and Utilization of Subtropical Agro-bioresources, Guangdong Province Key Laboratory of Microbial Signals and Disease Control, Integrative Microbiology Research Centre, South China Agricultural University, Guangzhou, China; ^2^Laboratory of Insect Toxicology, and Key Laboratory of Pesticide and Chemical Biology, Ministry of Education, South China Agricultural University, Guangzhou, China

**Keywords:** bioremediation, D-phenothrin, *Pseudomonas fulva*, metabolites, degradation pathway

## Abstract

D-phenothrin is one of the most popular pyrethroid insecticides for its broad spectrum and high insecticidal activity. However, continuous use of D-phenothrin has resulted in serious environmental contamination and raised public concern about its impact on human health. Biodegradation of D-phenothrin has never been investigated and its metabolic behaviors remain unknown. Here, a novel bacterial strain P31 was isolated from active sludge, which completely degraded (100%) D-phenothrin at 50 mg⋅L^-1^ in 72 h. Based on the morphology, 16S rRNA gene and Biolog tests, the strain was identified as *Pseudomonas fulva*. Biodegradation conditions were optimized as 29.5°C and pH 7.3 by utilizing response surface methodology. Strain P31 depicted high tolerance and strong D-phenothrin degradation ability through hydrolysis pathway. Strain P31 degraded D-phenothrin at inhibition constant (*K*_i_) of 482.1673 mg⋅L^-1^ and maximum specific degradation constant (*q*_max_) of 0.0455 h^-1^ whereas critical inhibitor concentration remained as 41.1189 mg⋅L^-1^. The 3-Phenoxybenzaldehyde and 1,2-benzenedicarboxylic butyl dacyl ester were identified as the major intermediate metabolites of D-phenothrin degradation pathway through high-performance liquid chromatography and gas chromatography-mass spectrometry. Bioaugmentation of D-phenothrin-contaminated soils with strain P31 dramatically enhanced its degradation, and over 75% of D-phenothrin was removed from soils within 10 days. Moreover, the strain illustrated a remarkable capacity to degrade other synthetic pyrethroids, including permethrin, cyhalothrin, β-cypermethrin, deltamethrin, fenpropathrin, and bifenthrin, exhibiting great potential in bioremediation of pyrethroid-contaminated environment.

## Introduction

D-phenothrin [(1*R-trans*)-2,2-dimethyl-3-(2-methyl-1-propenyl)-(3-phenoxyphenyl) methyl ester] is a type I synthetic pyrethroid (SP) insecticide which contains a cyclopropanecarboxylic acid group linked to an aromatic alcohol through a central ester bond (Supplementary Figure 1). D-phenothrin is widely used to control insects related to public health including mosquitoes, ticks, mites, human head lice and scabies, as well as insect pests of animals and agricultural crops (Scott et al., 2015). SP insecticides are analogs of pyrethrins found in *Chrysanthemum cinerariaefolium* flowers (Cycoń and Piotrowska-Seget, 2016). Applications of SPs have significantly increased over the past two decades because of their ability to paralyze the target insects and low mammalian toxicity as compared to organochlorines and organophosphates (Scott et al., 2015). Half-life of pyrethroids is usually less than 600 days and are generally considered safe for humans and animals (Laskowski, 2002), but pyrethroid residues are frequently found in groundwater, sediments, foods, and mammals (Delgado-Moreno et al., 2011; Kuivila et al., 2012; Markle et al., 2014). Pyrethroid bioaccumulation was first reported in wild river fish in Iberian river basins of Spain (Corcellas et al., 2015), and in 100% honeybee wax samples (García et al., 2017). Pyrethroid intoxication to endocrine activity causing estrogenic or antiestrogenic effects and immune responses in fish and mammals was also reported (Ansari et al., 2011; Saillenfait et al., 2015; Brander et al., 2016). In addition, D-phenothrin exposure to humans and mammals can lead to DNA damage *in vitro* (Nagy et al., 2014; Atmaca and Aksoy, 2015). Such serious environmental contaminations of SPs are not only effecting the wild life but human population as well. This situation demands for the advanced remediating strategies to tackle pyrethroid-polluted environments.

To remove pesticide residues from the environment, several remediation technologies such as photodecomposition, fenton degradation, ozonation, adsorption, incineration, and biodegradation have been developed (Arora et al., 2017; Cycoń et al., 2017; Morillo and Villaverde, 2017; Zhan et al., 2018a). Recently, bioremediation through bioaugmentation and/or biostimulation, has emerged as the most cost-effective and eco-friendly approach to breakdown pesticides in soils (Gao et al., 2012; Cycoń and Piotrowska-Seget, 2016). Different pyrethroid-degrading strains, such as *Sphingobium* sp. JZ-1 (Wang et al., 2009), *Stenotrophomonas* sp. ZS-S-01 (Chen et al., 2011d), *Ochrobactrum anthropic* YZ-1 (Zhai et al., 2012), *Brevibacterium aureum* DG-12 (Chen et al., 2013), *Serratia marcescens* (Cycoń et al., 2014), *Bacillus thuringiensis* ZS-19 (Chen et al., 2015), *Pseudomonas aeruginosa* GF31 (Tang et al., 2017), and *Acinetobacter baumannii* ZH-14 (Zhan et al., 2018b) have already been isolated from contaminated soils. However, microbial degradation of D-phenothrin and its pathway has never been investigated.

The objectives of this study were: (1) to investigate the microbial degradation of D-phenothrin and its biochemical degradation pathway; (2) to determine the biodegradation kinetics of D-phenothrin; and (3) to evaluate the potentials of isolate for bioremediation of D-phenothrin-contaminated environment.

## Materials and Methods

### Chemicals and Media

D-phenothrin (98% purity) was obtained from Jiangsu Yangnong Chemical Group Co., Ltd., China. Technical-grade permethrin, cyhalothrin, β-cypermethrin, deltamethrin, fenpropathrin, and bifenthrin were purchased from Sigma–Aldrich, United States and high-performance liquid chromatography (HPLC)-grade acetonitrile was purchased from Fisher Scientific, United States. All other chemicals and solvents were of analytical grade. SPs were dissolved in dimethyl sulfoxide (DMSO) or acetone at a stock concentration of 10 g⋅L^-1^, and stored in dark bottles at 4°C.

Mineral salt medium (MSM) containing (g⋅L^-1^) (NH_4_)_2_SO_4_, 2; MgSO_4_⋅7H_2_O, 0.2; CaCl_2_⋅2H_2_O, 0.01; FeSO_4_⋅7H_2_O, 0.001; Na_2_HPO_4_⋅12H_2_O, 1.5; and KH_2_PO_4_, 1.5; and Luria-Bertani (LB) medium containing (g⋅L^-1^) tryptone, 10; yeast extract, 5; and NaCl, 10 were used in this study. Both culture media were adjusted to pH 7.3 and sterilized at 121°C for 20 min.

### Isolation of D-Phenothrin-Degrading Bacterial Strains

Active sludge from an aerobic pyrethroid-manufacturing wastewater treatment system in Zhongshan, China, was used to achieve D-phenothrin-degrading bacterial isolates. Isolation and enrichment of degrading bacterial isolates was carried out according to Chen et al. (2011b,c). Initial culture enrichment was performed in a 250-mL Erlenmeyer flask containing 50 mL of sterilized MSM supplemented with 5 g of activated sludge and 50 mg⋅L^-1^ of D-phenothrin. Enrichment culture was incubated in an incubator shaker at 180 rpm and 30°C for 7 days. After 7 days, 0.5 mL of enrichment culture was transferred to new Erlenmeyer flasks containing 50 mL of fresh MSM and 100, 200, 400, and 800 mg⋅L^-1^ D-phenothrin, respectively. After five rounds of transferring, the enrichment medium was serially diluted and spread on MSM plates along with 50 mg⋅L^-1^ D-phenothrin to isolate individual colonies. After purifying the obtained isolates, their D-phenothrin degradation potential was investigated. Concentrations of D-phenothrin residues in culture fluids were detected by HPLC (Waters 2690, United States).

### Identification and Characterization of Strain P31

Colony and cellular morphologies of strain P31 were studied under electron microscope (BH-2 Olympus, Japan) and scanning electron microscope (XL-30 ESEM, Philips Optoelectronics Co., Ltd., Holland) on LB plates and slides, respectively.

Strain P31 was identified by sequencing 16S rRNA, which is the most conserved DNA region in prokaryotes (Festa et al., 2013). Genomic DNA was prepared by using MasterPure^TM^ DNA Purification Kit (Epicentre Biotechnologies, United States). 16S rRNA gene was amplified with reference (*Escherichia coli*) primers 27F (5V-AGA GTT TGA TCC TGG CTC AG-3V) and 1492R (5V-TAC GGY TAC CTT GTT ACG ACT T-3V) and NCBI BLAST tool was used to compare 16S rRNA sequences. Bacterium was further identified by Biolog Microbial Identification System (GP2 MicroPlate^TM^, BIOLOG, Hayward, CA, United States).

### Growth and Degradation Characteristics of Strain P31

Strain P31 was thawed and inoculated in LB medium for 12 h. Bacterial cells were harvested by centrifugation (5 min, 1950 × *g*) and washed twice with sterile saline (0.9% NaCl) to prepare a concentrated inoculum solution (Chen et al., 2011a). A total of 0.3 g⋅L^-1^ of inoculum was transferred to 50 mL of MSM medium containing 50 mg⋅L^-1^ of D-phenothrin, and kept at 29.5°C and 200 rpm in a rotary shaker. Initial OD_600_ of the culture was about 0.05. All set-ups were prepared in triplicates, and non-inoculated cultures served as controls. Samples were collected at 0, 12, 24, 36, 48, 60, and 72 h, respectively. Residual concentrations of D-phenothrin were detected by HPLC.

### Optimization of D-Phenothrin Degradation Conditions

Based on Box–Behnken design, optimized conditions of D-phenothrin biodegradation were determined according to the response surface methodology (RSM) (Ghevariya et al., 2011; Zhang et al., 2011; Chen et al., 2013). For statistical calculations, the variables *X*_i_ (the uncoded value of the *i*th independent variable) were coded as *x*_i_ (the coded value of the *i*th independent variable) according to the following equation:

$$x_{i}=(X_{i}-X_{0})/\Delta X_{i}$$

where *x*_i_ is the value of *X*_i_ at the center point, and Δ*X*_i_ is the step change value.

Based on the preliminary results of one-factor-at-a-time experiments (data not shown), critical factors such as temperature, pH and inoculum size were selected as independent variables. Levels of the variables and experimental Box–Behnken design are shown in Supplementary Table 1. Accordingly, based on Box–Behnken design 15 experiments were carried out to build quadratic models, with three replications of the center points to estimate experimental error. Experimental data of the model experiments was used to predict optimal conditions according to the following equation:

$$Y_{i}=b_{0}+\Sigma {ϸ}_{i}X_{i}+\Sigma {ϸ}_{ij}X_{i}X_{j}+\Sigma {ϸ}_{ii}X_{ii}^{2}+e_{i}$$

where *X*_i_ are the input variables, which influence the response variable *Y*_i_, ϸ_0_ is the offset term, ϸ_i_, ϸ_ii_, and ϸ_ij_ are the first-order, quadratic, and interaction coefficients, respectively, *i* and *j* are the index numbers for the factors, and e_i_ is the residual error.

SAS 9.0 was used for statistical analysis and building regression model to predict optimal processing parameters.

### Dynamics and Substrate Range Characteristics of Strain P31

Degradation dynamics parameters were determined by measuring specific degradation constant at various D-phenothrin concentrations (25, 50, 100, 200, 400, and 800 mg⋅L^-1^) under optimal reaction conditions. D-phenothrin residual concentrations were detected and specific degradation constant (*q*) at different initial concentrations was assessed according to substrate inhibition model (Luong, 1987), as follows.

$$q=(q_{\max}S)/S+K_{s}+\left(S^{2}/K_{i}\right)$$

where *q*_max_ is the maximum specific degradation constant, *K*_s_ is the half-saturation constant, *K*_i_ is the substrate inhibition constant, and *S* is the inhibitor concentration. Kinetic parameters were calculated by non-linear regression method in MATLAB 7.8.

To assess the degradation ability of strain P31 against various SPs, MSM medium was supplemented with D-phenothrin, permethrin, cyhalothrin, β-cypermethrin, deltamethrin, fenpropathrin, or bifenthrin at 50 mg⋅L^-1^ along with the optimal inoculum size. Each treatment was performed in triplicate and incubated at optimum conditions whereas non-inoculated samples served as control. Samples of different treatments were collected at regular intervals and degradation was measured as described above.

### Identification of D-Phenothrin Metabolic Products

To identify D-phenothrin and its metabolic products of biodegradation, the strain P31 was grown in MSM media containing 50 mg⋅L^-1^ of D-phenothrin. Non-inoculated samples containing the same amount of D-phenothrin served as control. Samples of different treatments were collected at regular intervals as described above, and extracted after acidification to pH 2 (2 M HCl) according to Tallur et al. (2008). Metabolites were detected by gas chromatography-mass spectrometry (GC-MS) (Agilent 6890N/5975, United States). To identify the metabolites, mass spectrometry analyses were matched with authentic standard compounds from the National Institute of Standards and Technology (NIST, United States) library database.

### Biodegradation of D-Phenothrin in Soils

Soil samples were collected at the depth of 5–20 cm from a field, which had never been treated with D-phenothrin or fertilizers, in South China Agricultural University, Guangzhou, China. Physicochemical properties of the soils were as (g/kg of dry weight): organic matter, 10.5; total N, 0.5; total P, 0.4; total K, 18.2; and pH, 6.9 and has sandy loam texture (sand, 65.0%; silt, 28.0%; clay, 7.0%). Soil samples were air-dried in the laboratory to the suitable moisture level, sieved (2 mm), and used for bioremediation studies (Chen et al., 2012, 2014).

Sterile (at 121°C for 1 h) and nonsterile soils (nSSs) were used to investigate bioremediation ability of strain P31. A total of 600 g of sterile or nSSs were placed in 500-mL Erlenmeyer flasks, and D-phenothrin solution was added to a final concentration of 50 mg⋅kg^-1^ of soil in acetone solution. Erlenmeyer flasks were kept opened to allow aerobic conditions. Water contents were adjusted to 40% of water-holding capacity with deionized water (Cycoń et al., 2014). After mixing, the strain P31 suspension was introduced into the soils by drip irrigation to a final concentration of 1.0 × 10^7^ CFU⋅g^-1^ of soil and incubated in a sterile incubator at 30°C. Sterile or nSS samples having insecticides but without strain P31 suspension were used as controls. Triplicate soil samples were kept in a dark thermostatic chamber for 10 days at 30°C.

Soil samples (20 g) for D-phenothrin residue analysis were collected on day 0, 2, 4, 6, 8, and 10. Samples were ultrasonically extracted with 40 mL of ethyl acetate for 15 min, and then centrifuged at 4000 × *g* for 6 min to obtain supernatants. Supernatants were pooled and loaded onto a chromatography column (35 cm length × 1 cm i.d., pre-eluted with 40 mL of ethyl acetate), packed with anhydrous sodium sulfate, silica gel, and neutral alumina. Column was eluted three times, with 60 mL of ethyl acetate. Eluates were evaporated and residue was re-dissolved in 1.5 mL methanol for HPLC analysis.

Degradation constant (*k*) was determined according to the first-order kinetic model (Eq. 4) (Cycoń et al., 2009):

$$C_{t}=C_{0}\times e^{-kt}\,\;\left(4\right)$$

where *C*_0_ is the initial concentration of D-phenothrin at zero time, *C*_t_ is the concentration of D-phenothrin at time *t*, *k*, and *t* are the degradation constant (day^-1^) and degradation period in days, respectively.

D-phenothrin half-life (*t*_1/2_) in different soils was calculated by using Eq. 5:

$$t_{1/2}=In\,\;2/k$$

where *k* is the degradation constant (day^-1^).

### Chemical Analysis

Analysis of D-phenothrin residues in filtrates was carried on a Phenomenex C_18_ reversed phase column (250 × 4.60 mm, 5 μm) with a Water 2690 HPLC system. A mixture of acetonitrile and water (90:10) was used as the mobile phase at the flow rate of 1.0 mL⋅min^-1^. Based on retention time (RT) and peak area of pure standard, array detection was carried out from 190 to 400 nm (total scan) at detection limit of 0.01 mg⋅L^-1^. Detection wavelengths of D-phenothrin, permethrin, cyhalothrin, β-cypermethrin, deltamethrin, fenpropathrin, and bifenthrin were as 276.8, 276.8, 276.8, 276.8, 250.9, 276.8, and 354 nm, respectively.

Analysis of D-phenothrin metabolic products, was performed on Agilent 6890N/5975 GC-MS system equipped with auto-sampler, an on-column, split/splitless capillary injection system with array detection from 30 to 500 nm (total scan). Chromatographic column was a HP-5MS capillary column (30.0 m × 250 μm × 0.25 μm), and column temperature was initially held at 90°C for 2 min, then increased to 150°C at the rate of 6°C⋅min^-1^ for 1 min, raised to 180°C at the rate of 10°C⋅min^-1^ for 4 min and finally increased to 260°C at the rate of 20°C⋅min^-1^ for 10 min. Carrier gas (helium) flow rate was 1.5 mL⋅min^-1^ and the injection volume was 1.0 μL with splitless sampling at 250°C. Temperatures corresponding to transfer line and the ion trap were 280°C and 230°C, respectively, and the ionization energy was 70 eV (Chen et al., 2014, 2015).

### Nucleotide Sequence Accession Number

The GenBank accession number of strain P31 16S rRNA gene is KY229868.

## Results

### Isolation of D-Phenothrin-Degrading Strain

Based on the isolation and enrichment results, 34 morphologically different strains (grown on MSM containing 800 mg⋅L^-1^ D-phenothrin) were attained from activated sludge and named as P01∼34, respectively. Results showed that strain P31 possessed the highest degradation capacity among 34 strains and degraded 100% of D-phenothrin within 72 h of incubation. Degradation of D-phenothrin by each isolated strain within 72 h of incubation is shown in Supplementary Table 2. Moreover, strain P31 was also found to efficiently utilize permethrin, cyhalothrin, β-cypermethrin, deltamethrin, fenpropathrin, and bifenthrin as the sole source of carbon. Thus, strain P31 was selected for further studies.

### Identification and Characterization of Strain P31

Physio-biochemical properties of the strain P31 are shown in Supplementary Table 3. Strain P31 is a Gram-negative, obligatory aerobic and rod-shaped bacterium of 1.5∼4.0 μm length and 0.5∼1.0 μm width. Colonies grown on LB agar plates were primrose yellow, opaque, and circular with smooth margin. This bacterial isolate showed positive reactions in catalase, oxidase, citrate utilization, nitrate reduction, hemolysis, and arginine dihydrolase tests, but remained negative in anaerobic growth, gelatin liquefaction, urease production, indole production, and esculin hydrolysis tests. Growth was observed over a temperature range of 20∼40°C, a salinity range of 2∼5% NaCl, and a pH range of 5∼10. It was positive in the utilization of glycerol, α-D-glucose, D-fructose, L-fucose, L-alanine, dextrin, L-serine, inosine, D-galactose, D-fucose, methyl pyruvate, sodium bromate; and negative in the utilization of sucrose, pectin, gelatin, D-maltose, D-trehalose, D-cellobiose, stachyose, D-turanose, gentiobiose, D-raffinose, D-melibiose, and β-methyl-D-glucoside.

According to BLAST analysis, partial sequence showed high similarity to 16S rRNA gene sequence of *Pseudomonas* group and closely clustered with strain f2 (2012) (GenBank accession No. JQ717288) and strain SRZ19 (GenBank accession No. KU877341), having sequence identities over 99%. Similarity and distance between strain P31 and the model strain (*P. fulva*) were 0.617 and 4.355, respectively, according to Biolog microbial identification system. Therefore, the strain P31 was confirmed as *P. fulva* based on the morphology, physio-biochemical properties, 16S rRNA gene analysis, and Biolog microbial identification system.

### Growth and Degradation Characteristics of Strain P31

**FIGURE 1:**
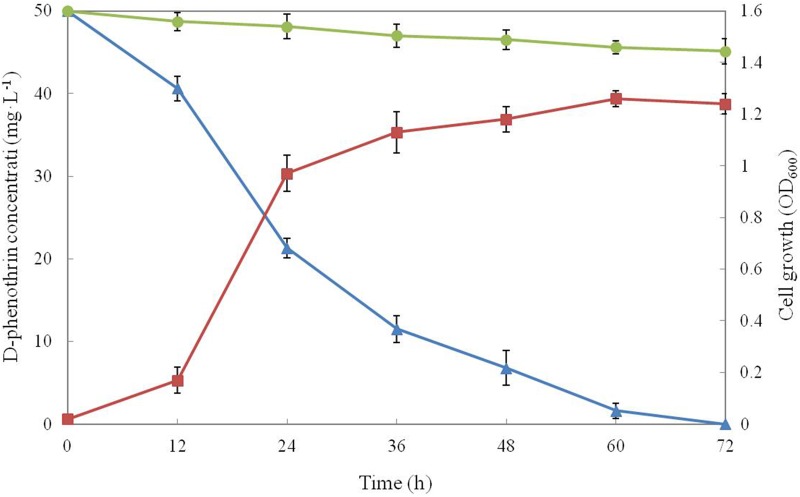
Biodegradation of D-phenothrin (50 mg⋅L^-1^) during growth of strain P31. Symbol: •, D-phenothrin control; 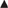, D-phenothrin degradation by strain P31; 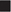, cell growth. Data represent mean values of three replicates with standard deviation.

Growth of strain P31 in MSM media with D-phenothrin as the sole carbon resource and its degradation characteristics are shown in **Figure 1**. Strain P31 showed rapid growth without lag phase during initial period (0∼12 h) indicating that the strain utilized D-phenothrin as growth substance. Cell growth hiked after 12 h of incubation, and significant degradation of D-phenothrin was noted during this period. Cell growth increased rapidly at logarithmic phase (12∼24 h), and approximately 57.0% of the D-phenothrin was degraded by strain P31 in 24 h. During this time, the concentration of D-phenothrin decreased with a concomitant increase of cell number. Subsequently, cell growth slowed down at stationary phase (36∼60 h), and about 77.0–86.4% of the initial dose was eliminated within 36–48 h, respectively. Strain P31 reached to maximum level of growth at 60 h post inoculation and caused the maximum D-phenothrin biodegradation of 96.8%. Finally, the strain did not show growth at decline phase (> 72 h) and the residual amount of D-phenothrin was not detectable by HPLC at 72 h post incubation. Approximately 9.0% degradation was observed in the non-inoculated control.

### Optimization of D-Phenothrin Degradation Conditions

Supplementary Table 1 demonstrates the level and range of three independent variables, including temperature (*X*_1_), pH (*X*_2_), and inoculum size (*X*_3_). The three parameters were taken at a central coded value considered as zero and investigated at three different levels (-1, 0, and +1). In this case, the result of a three-level factorial design, a total number of 15 experiments were employed to match the second-order polynomial model in accordance with Box–Behnken design. Statistical combinations of critical parameters along with the maximum observed and predicted degradation are displayed in Supplementary Table 1. These predicted values were in close proximity to the observed ones in all sets of experiments. Highest degradation of 100% and the lowest of 80.4% were observed. Two regression equations, Eq. 6a for coded values and Eq. 6b for actual experimental values, which are analogous to Eq. 2, showed the degradation rate (*Y*) as a function of the test variables temperature (*X*_1_), pH (*X*_2_), and inoculum size (*X*_3_).

$$Y=100-1.9375X_{1}+1.35X_{2}+2.7375X_{3}-9.4875X_{1}^{2}+0.2X_{1}X_{2}-0.075X_{1}X_{3}-5.5625X_{2}^{2}+1.45X_{2}X_{3}-1.1375X_{3}^{2}$$

$$Y=-494.163+22.1325X_{1}+75.125X_{2}-24.125X_{3}-0.3795X_{1}^{2}+0.04X_{1}X_{2}-0.15X_{1}X_{3}-5.5625X_{2}^{2}+14.5X_{2}X_{3}-113.75X_{3}^{2}$$

Analysis of variance (ANOVA) is essential to test the significance and adequacy of the model (Ghevariya et al., 2011) and ANOVA of the quadratic regression model is tabulated in Supplementary Table 4. As shown in the table, a very low probability value (*P*-value < 0.05), a large value of the regression coefficient (*R*^2^ = 0.9905) and a low coefficient of variation (CV = 1.10) demonstrated that the established quadratic polynomial model for D-phenothrin degradation by strain P31 presented the actual relationship of response and variables. Regression analysis reveals that the linear terms of temperature (*X*_1_), pH (*X*_2_), and inoculum size (*X*_3_) are important factors in the D-phenothrin degradation by strain P31. Square terms of *X*_1_^2^ and pH *X*_2_^2^, interaction terms of *X*_1_*X*_3_ and *X*_2_*X*_3_ also showed significant effects (*P* < 0.05) on D-phenothrin degradation by strain P31, however, square terms of *X*_3_^2^ and interaction term of *X*_1_*X*_2_ played insignificant role (*P* > 0.05) in degradation (Supplementary Table 4).

For better understanding of the results, a 3D response surface plot is given in Supplementary Figure 2, which illustrates the effects of temperature (*X*_1_) and pH (*X*_2_) on D-phenothrin degradation with inoculum size (*X*_3_) as a constant. Supplementary Figure 2 has clear stationary point, indicating that maximum degradation could be achieved inside the design boundaries. Model depicted maximum degradation of 100% at temperature 29.5°C, pH 7.3 and an inoculum size of 0.3 g⋅L^-1^. Thus, the optimum culturing conditions for D-phenothrin degradation by strain P31 were concluded to be temperature 29.5°C, pH 7.3 and an inoculum size of 0.3 g⋅L^-1^.

### Biodegradation Kinetics of D-Phenothrin by Strain P31

**FIGURE 2:**
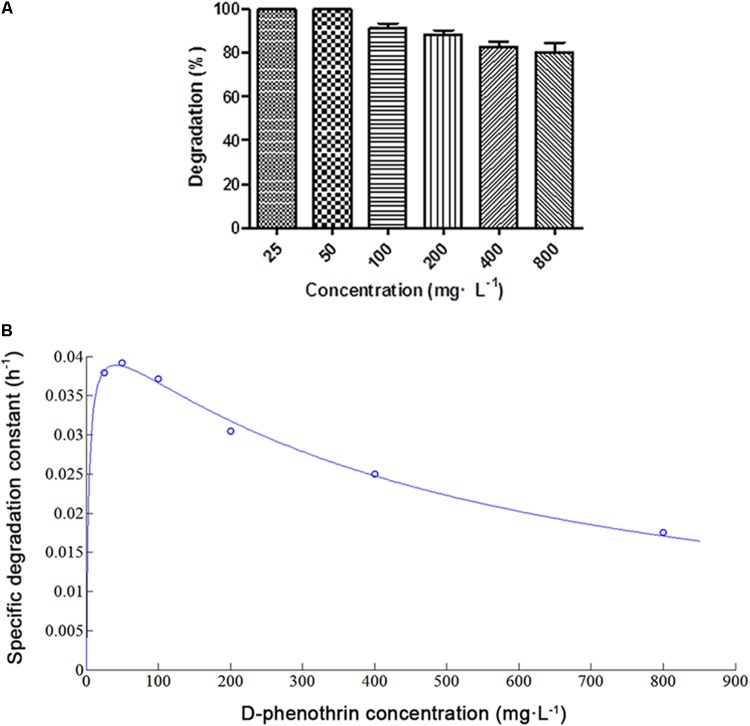
Biodegradation of D-phenothrin at different initial concentrations by strain P31 within 72 h. **(A)** Degradation of D-phenothrin at different concentrations; **(B)** Relationship between initial D-phenothrin concentration and specific degradation constant by strain P31. Data represent mean values of three replicates with standard deviation.

D-phenothrin biodegradation at different initial D-phenothrin concentrations is presented in **Figure 2A**. Strain P31 utilized and degraded D-phenothrin up to a concentration of 800 mg⋅L^-1^, and no lag period was observed. D-phenothrin was degraded completely within 72 h at initial concentrations below 50 mg⋅L^-1^. During 72 h incubation, strain P31 displayed 91.2, 88.3, 82.6, and 80.4% of degradation at D-phenothrin initial concentrations of 100, 200, 400, and 800 mg⋅L^-1^, respectively. Increased concentration of D-phenothrin had a slight effect on biodegradation performance of strain P31, indicating that the D-phenothrin degradation by this strain is concentration dependent. Relationship between initial D-phenothrin concentration and specific degradation constant by strain P31 is shown in **Figure 2B**. According to the substrate inhibition model (Eq. 3), *K*_s_ and *K*_i_ values were determined by non-linear regression method as 3.5066 and 482.1673 mg⋅L^-1^, respectively. Critical inhibitor concentration (*S_m_*) was calculated as 41.1189 mg⋅L^-1^ whereas the overall *q*_max_ value remained 0.0455 h^-1^. As shown in **Figure 2B**, at the initial concentration of D-phenothrin below 50 mg⋅L^-1^, *q* value was rapidly increased. At the initial concentrations of D-phenothrin above 100 mg⋅L^-1^, *q* value gradually declined with the increase of D-phenothrin. These results illustrated that the biodegradation of D-phenothrin by strain P31 could be partially inhibited at high concentration of D-phenothrin.

### Biodegradation Kinetics of Various SPs

**FIGURE 3:**
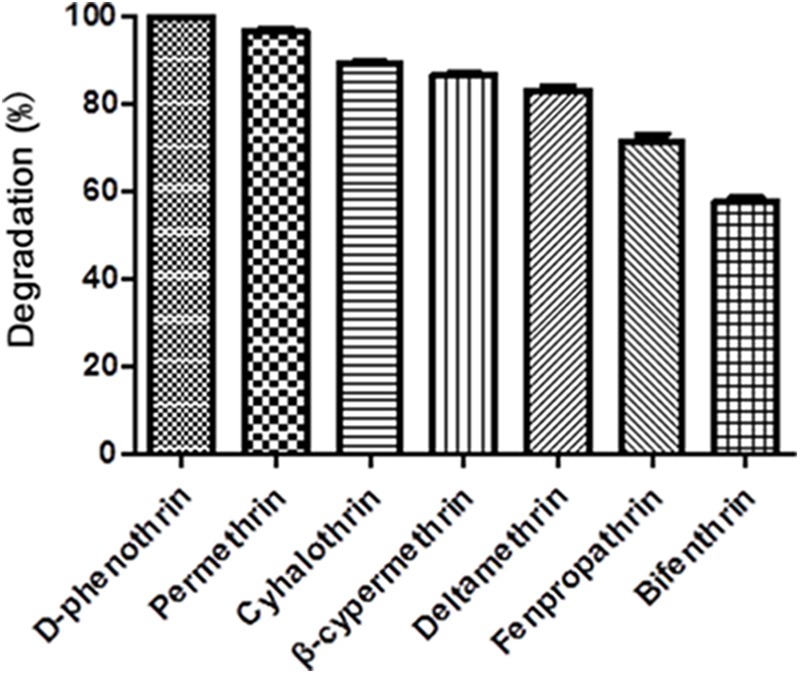
Degradation of various synthetic pyrethroids by strain P31 within 72 h. Data represent mean values of three replicates with standard deviation.

Strain P31 revealed capability to utilize various SPs as food source including D-phenothrin, permethrin, cyhalothrin, β-cypermethrin, deltamethrin, fenpropathrin, and bifenthrin, having degradation efficiency of 100, 96.7, 89.2, 86.6, 83.1, 71.4, and 57.9% within 72 h, respectively (**Figure 3**). To further quantify the biodegradation capacity of strain P31 against different SPs, the degradation constant (*k*) and half-life (*t*_1/2_) were determined by using the first-order kinetic model. Degradation kinetic parameters of various SPs by strain P31 are summarized in **Table 1**. Biodegradation process was characterized by a *k* ranging from 0.0117 to 0.0392 h^-1^. The *t*_1/2_ value of the above mentioned SPs with strain P31 were calculated as 17.7, 21.0, 23.7, 25.9, 27.4, 36.9, and 59.2 h, respectively, and are significantly lower as compared to those in the environment.

**Table 1:** Kinetic parameters of degradation of synthetic pyrethroids by strain P31.

Synthetic pyrethroids	Regression equation	*k* (h^-1^)	*t*_1/2_ (h)	*R*^2^
D-phenothrin	*C*_t_ = 53.5150e^-0.0392^*^t^*	0.0392	17.7	0.9802
Permethrin	*C*_t_ = 53.8361e^-0.0330^*^t^*	0.0330	21.0	0.9609
Cyhalothrin	*C*_t_ = 53.1980e^-0.0292^*^t^*	0.0292	23.7	0.9848
β-cypermethrin	*C*_t_ = 53.3589e^-0.0268^*^t^*	0.0268	25.9	0.9822
Deltamethrin	*C*_t_ = 52.1891e^-0.0253^*^t^*	0.0253	27.4	0.9736
Fenpropathrin	*C*_t_ = 57.7978e^-0.0188^*^t^*	0.0188	36.9	0.9902
Bifenthrin	*C*_t_ = 53.7381e^-0.0117^*^t^*	0.0117	59.2	0.9687


### Metabolic Products and Degradation Pathway of D-Phenothrin

**FIGURE 4:**
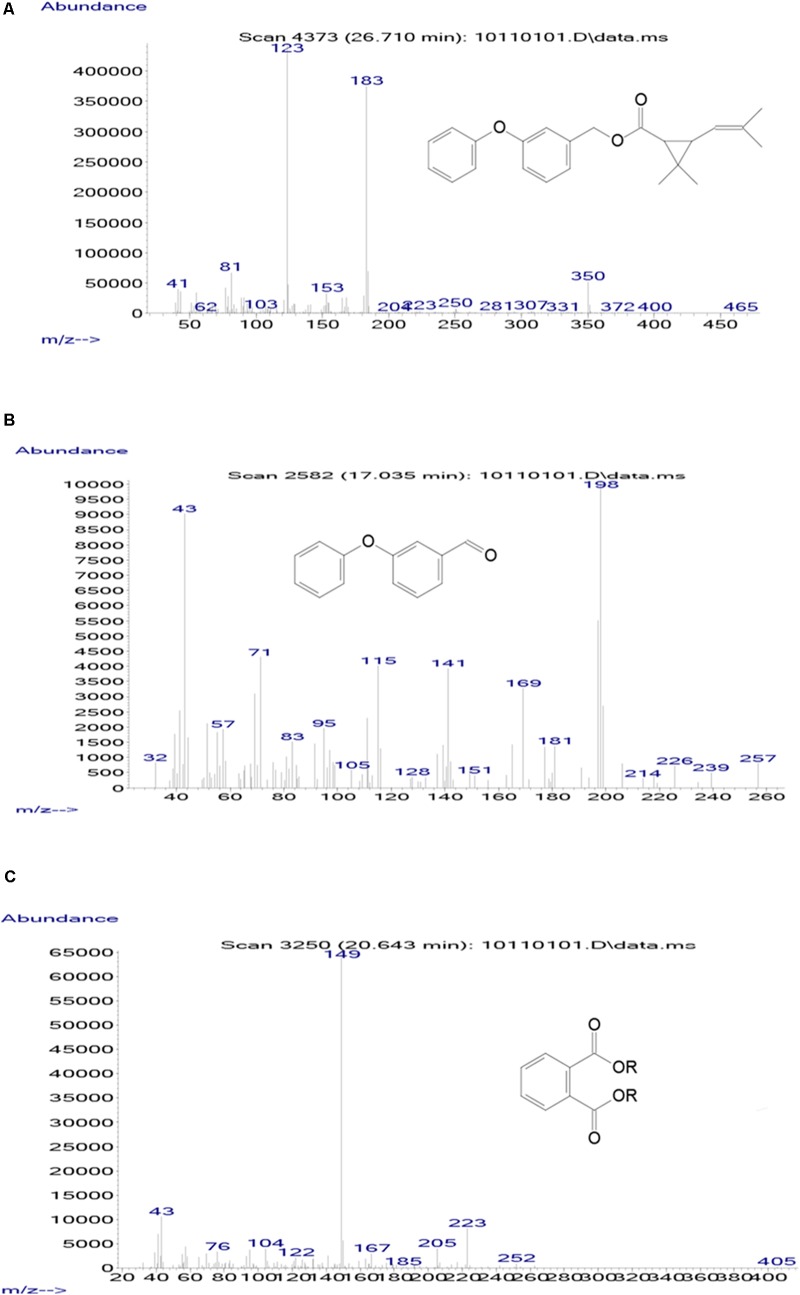
GC-MS analysis of the metabolites produced from D-phenothrin degradation by strain P31. **(A–C)** The characteristic ions of compounds A–C in GC-MS. Retention time of each compound was 26.710, 17.035, and 20.643 min, respectively, and identified as D-phenothrin, 3-phenoxybenzaldehyde, and 1,2-benzenedicarboxylic butyl dacyl ester, respectively.

Samples obtained at different intervals were analyzed through GC-MS to identify the metabolic products during the bacterial degradation process. In the initial sample of 0 and 12 h, a significant peak was detected at RT of 26.710 min, displaying a characteristic mass fragment [M^+^] at *m*/*z* 350 with major fragment ions at *m*/*z* 123 and 183, which was identified as D-phenothrin and named as compound A. Subsequently, the compound A disappeared concomitantly with the formation of two new compounds. According to the similarity of their fragment RTs and molecular ions to those of corresponding authentic compounds in the NIST library database, the two compounds were characterized as 3-phenoxybenzaldehyde (compound B) and 1,2-benzenedicarboxylic butyl dacyl ester (compound C), respectively (Supplementary Figure 3). It is the first time that these metabolic products were detected in the degradation pathway of D-phenothrin. It is noteworthy that all these metabolites were transitory and faded away without any non-cleavable metabolites at the end of experiment. Chemical structures, RT, and characteristic ions of the mass spectra are shown in **Figure 4**.

**FIGURE 5:**
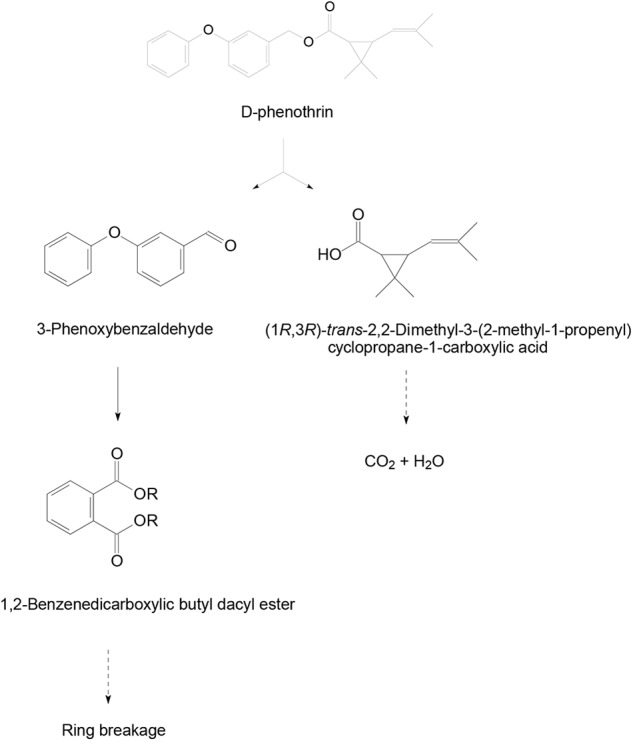
Proposed degradation pathway of D-phenothrin in strain P31.

A degradation pathway of D-phenothrin in strain P31 was proposed based on the chemical structures of D-phenothrin and metabolites formed during fermentation (**Figure 5**). D-phenothrin was first hydrolyzed by cleavage of the carboxylester linkage to produce 3-phenoxybenzaldehyde and (1*R*,3*R*)-trans-2,2-dimethyl-3-(2-methyl-1-propenyl) cyclopropane-1-carboxylic acid. Then, the intermediate product 3-phenoxybenzaldehyde was further degraded with diaryl cleavage to form 1,2-benzenedicarboxylic butyl dacyl ester. Eventually, D-phenothrin was degraded by strain P31 without any persistent accumulative product.

### Biodegradation of D-Phenothrin in Soils

**FIGURE 6:**
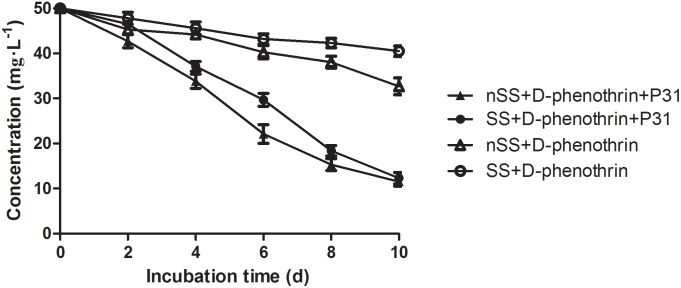
Degradation kinetics of D-phenothrin in sterile and nSSs. Data represent mean values of three replicates with standard deviation. SS refers to sterile soils; nSS refers to nonsterile soils.

The ability of strain P31 to degrade D-phenothrin in contaminated soils was evaluated under controlled conditions (**Figure 6**). Degradation process was further investigated using the first-order kinetic model as shown in **Table 2**. D-phenothrin degraded rapidly after strain P31 inoculation (1 × 10^7^ CFU⋅g^-1^ of soil) in nSS at the beginning of incubation, and approximately 77.0% of D-phenothrin at initial concentration of 50 mg⋅kg^-1^ was removed within 10 days without any lag phase. Degradation process was characterized by a degradation constant (*k*) of 0.1380 day^-1^ and *t*_1/2_ value of 5.0 days (**Table 2**). By contrast, only 34.6% of added pesticide was degraded in the control group inoculated with autochthonous microorganisms, at *t*_1/2_ value of 18.5 days. About 75.4% of initial D-phenothrin was removed from the sterile soil (SS) within 10 days at *k*-value of 0.1167 day^-1^ and *t*_1/2_ of 5.9 days. In the non-inoculated control, this activity decreased to only 19.1% in SS at *t*_1/2_ of 32.7 days. It is noteworthy that the degradation of D-phenothrin in nSS was higher as compared to SS.

**Table 2:** Kinetic parameters of degradation of D-phenothrin in sterile and nonsterile soils.

Soil treatments	Regression equation	*k* (day^-1^)	*t*_1/2_ (days)	*R*^2^
SS+D-phenothrin	*C*_t_ = 49.7988e^-0.0212^*^t^*	0.0212	32.7	0.9905
nSS+D-phenothrin	*C*_t_ = 50.0541e^-0.0374^*^t^*	0.0374	18.5	0.9592
SS+D-phenothrin+P31	*C*_t_ = 53.9713e^-0.1167^*^t^*	0.1167	5.9	0.9322
nSS+D-phenothrin+P31	*C*_t_ = 52.6082e^-0.1380^*^t^*	0.1380	5.0	0.9705


*SS refers to sterile soils; nSS refers to nonsterile soils.*

## Discussion

*Pseudomonas* bacterial strains are known for their metabolic capability, environmental versatility and ability to degrade different xenobiotic compounds including permethrin (Maloney et al., 1988), 3-phenoxybenzoate (Topp and Akhtar, 1991), β-cyfluthrin (Saikia et al., 2005), cypermethrin (Jilani and Khan, 2006), diazinon (Cycoń et al., 2009), 4-chloro-2-nitrophenol (Arora and Bae, 2014), 4-chloro-3-nitrophenol (Arora et al., 2014), fenpropathrin (Song et al., 2015), and β-cypermethrin (Zhang et al., 2011; Tang et al., 2017). *Pseudomonas fulva*, one of the highly potent degrading microbes of genus *Pseudomonas*, is a ubiquitous environmental bacterium. Nevertheless, the potential use of *P. fulva* in biodegradation of organic compounds has not received the attention it deserves. Our studies demonstrated that *P. fulva* can utilize various organic pollutants as food source and thus it can be successfully colonized in nutrient deficient niches.

Generally, it is considered that the screening and enrichment of environmental microorganisms is of great importance for selecting desirable degrading bacteria (Yang et al., 2011; Liu et al., 2015; Xiao et al., 2015). In this study, 34 morphologically different strains, which grew well in MSM with D-phenothrin as the sole carbon source, were selected from pyrethroid-contaminated active sludge through enrichment culture technique, and the most active bacterium strain P31 was chosen for further studies. This is the first time that a D-phenothrin-degrading strain P31 was isolated which completely degraded D-phenothrin in 72 h. Unlike other environmental bacteria, *P. fulva* P31 has remarkable capacity to degrade a variety of SPs including D-phenothrin, permethrin, cyhalothrin, β-cypermethrin, deltamethrin, fenpropathrin, and bifenthrin which are widely used insecticides and are associated with environmental contamination. Study reveals that this isolate has great potential as a degrader in cleaning-up pyrethroid residues from the environment (Delgado-Moreno et al., 2011; Kuivila et al., 2012; Markle et al., 2014).

The RSM provides reliable information for the optimization of key process parameters to enhance biodegradation by employing polynomial equation of empirical model to experimental data (Bezerra et al., 2008). Box–Behnken design has great advantages as it requires fewer experimental runs to efficiently estimate quadratic surfaces for the optimization of responses (Ghevariya et al., 2011). During this study, RSM based on Box–Behnken design was successfully explored to improve the biodegradation process in strain P31, and optimum D-phenothrin biodegradation conditions were determined as 29.5°C, pH 7.3, and an inoculum size of 0.3 g⋅L^-1^. In addition, two mathematical models (Eqs. 6a and 6b) could be used to predict and optimize D-phenothrin degradation conditions by strain P31 within given parameters. Models indicated maximum degradation rate of 100% under the optimum conditions. Similar enhanced degradation through RSM has been reported in a variety of degrading microbes such as *Achromobacter xylosoxidans* CG542 (Ghevariya et al., 2011), *Pseudomonas aeruginosa* CH7 (Zhang et al., 2011), *Brevibacterium aureum* DG-12 (Chen et al., 2013), and *Bacillus subtilis* BSF01 (Xiao et al., 2015).

Biodegradation process is often inhibited by high concentration of substrates, which limits their use in bioremediation of variable environments. Previous studies reported that the metabolic activity of pyrethroid-degrading microbes lead to complete catabolite repression at high substrate concentrations < 200 mg⋅L^-1^ (Jilani and Khan, 2006; Lin et al., 2011). Contrarily, increased concentrations of D-phenothrin did not cause much effect on the biodegradation performance of strain P31, and more importantly it did not inhibit cell growth (**Figure 2B**). It is noteworthy that the isolate tolerated and rapidly degraded D-phenothrin up to a concentration, as high as 800 mg⋅L^-1^, at *K*_i_ and *q*_max_ of 482.1673 mg⋅L^-1^ and 0.0455 h^-1^, respectively. This feature provides significant advantage to survive under various environments and utilize even the high concentrations of xenobiotic (Chen et al., 2013, 2014).

Hydrolysis plays a significant role in the biodegradation of SPs, which could be attributed to the presence of cyclopropane carboxylic acid moieties connected to aromatic alcohols with a central ester (or ether) bond in SPs. Ester bond is generally considered susceptible to degrading microbes (Stok et al., 2004; Heidari et al., 2005; Tallur et al., 2008; Wang et al., 2009; Zhang et al., 2011; Chen et al., 2015; Tang et al., 2017). In this study, D-phenothrin was initially hydrolyzed via cleavage of the ester bond to yield the main intermediate products 3-phenoxybenzaldehyde and 1,2-benzenedicarboxylic butyl dacyl ester (**Figure 4**). The 3-Phenoxybenzaldehyde is a common product of type II SPs, as also was detected in the biodegradation process of fenvalerate (Chen et al., 2011b), deltamethrin (Chen et al., 2011c), fenpropathrin (Chen et al., 2014), and β-cypermethrin (Xiao et al., 2015; Tang et al., 2017). However, this metabolite has been rarely observed in type I SPs. Involvement of metabolite 1,2-benzenedicarboxylic butyl dacyl ester in SPs biodegradation has never been reported. It is worth noting that the intermediate products were transient and disappeared gradually, without further accumulation of metabolites at the end of experiment, suggesting that the isolate may harbor a complete metabolic pathway for the degradation and metabolism of D-phenothrin. Based on metabolites analysis, a degradation pathway of D-phenothrin in strain P31 has been proposed during this study (**Figure 5**). The proposed D-phenothrin degradation pathway may provide new insights into the biogeocycle of this pesticide.

During this study, potential of the strain P31 for bioremediation of pyrethroid-contaminated soils was investigated under laboratory conditions. Bioaugmentation of D-phenothrin-contaminated soils with strain P31 significantly enhanced the degradation of D-phenothrin, and 75.4∼77.0% of the initial dose of D-phenothrin (50 mg⋅kg^-1^) was removed from soils within 10 days at *t*_1/2_ value of 5.0∼5.9 days; while in the control group without strain P31 it took 18.5∼32.7 days. Results indicate biodegradation as the primary mechanism of SPs dissipation in the environment whereas its abiotic breakdown is less important (Khan et al., 1988; Maloney et al., 1988). Notably, at a final bacterial count of 1 × 10^7^ cells⋅g^-1^ of soil, strain P31 rapidly degraded D-phenothrin in the beginning of incubation without any lag phase. These results contradict the findings of Cycoń et al. (2014) who reported the slow degradation of deltamethrin during initial 14 days, in the soil bioaugmented with *Serratia marcescens* DeI-1 and DEI-2 and only 10∼14% of the initial dose was removed. Usually, the introduced bacteria need to adapt to the soil conditions, xenobiotics and to the proliferation of autochthonous microorganisms utilizing this pollutant as a carbon and energy source (Zhang et al., 2012; Cycoń and Piotrowska-Seget, 2016). Unlike other bacterial isolates, *P. fulva* P31 has an exceptional ability to adapt and thrive in nutrient deficient ecological niches that makes it a potent strain for various applications.

## Conclusion

D-phenothrin-degrading bacterial strain *P. fulva* P31 was isolated and characterized in terms of its physiology, biochemistry, and biodegradation ability. Strain P31 revealed remarkable capacity to degrade a wide range of SPs that cause environmental contaminations. Based on metabolites analysis, we also proposed a D-phenothrin degradation pathway in strain P31. Moreover, this particular strain exhibited great advantages in bioremediation of pyrethroid-contaminated soils because of its adaptability in water and soil environments. These insights will facilitate to develop new strategies to curb pesticide residues related environmental pollution.

## Author Contributions

SC designed the experiments; JY, YF, HZ, KZ, and SC performed the experiments; JY, FY, and SC performed the data analysis; LZ provided the scientific expertise; JY, JL, FY, and SC wrote the manuscript.

## Conflict of Interest Statement

The authors declare that the research was conducted in the absence of any commercial or financial relationships that could be construed as a potential conflict of interest.
