# Supplementary material for: Characterization of a Pyrethroid-Degrading Pseudomonas fulva Strain P31 and Biochemical Degradation Pathway of D-Phenothrin
Source: Front Microbiol. 2018 May 16;9:1003. doi: 10.3389/fmicb.2018.01003 (PMC5964208; doi:10.3389/fmicb.2018.01003)
Supplement: Supplementary file 1 [file Data_Sheet_1.PDF]

Supplementary Material

Characterization of a pyrethroid-degrading *Pseudomonas fulva* strain P31 and biochemical degradation pathway of D-phenothrin

Jingjing Yang[#], Yanmei Feng[#] , Hui Zhan, Jie Liu, Fang Yang, Kaiyang Zhang, Lianhui Zhang, Shaohua Chen*

*** Correspondence:** Shaohua Chen: shchen@scau.edu.cn

[#] Both authors contributed equally to this work.

Supplementary Figures

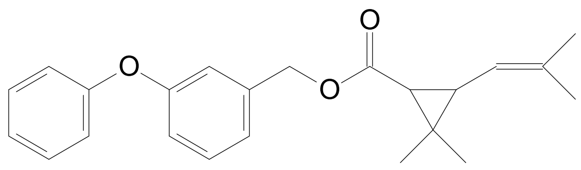

Supplementary Figure 1. Molecular structure of D-phenothrin

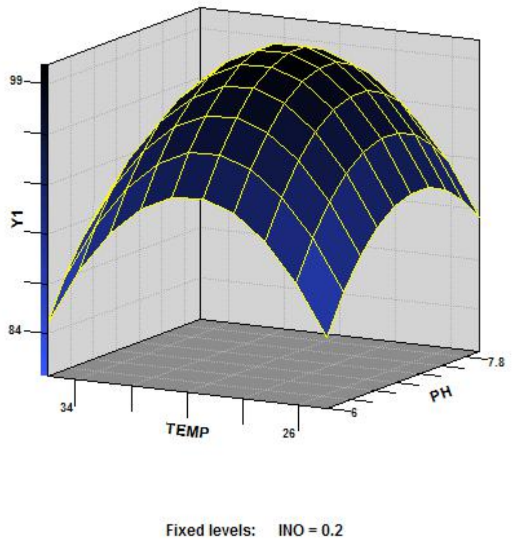

Supplementary Figure 2. Response surface plot showing the effect of temperature and pH on D-phenothrin degradation by strain P31 while fixing the value of inoculum size

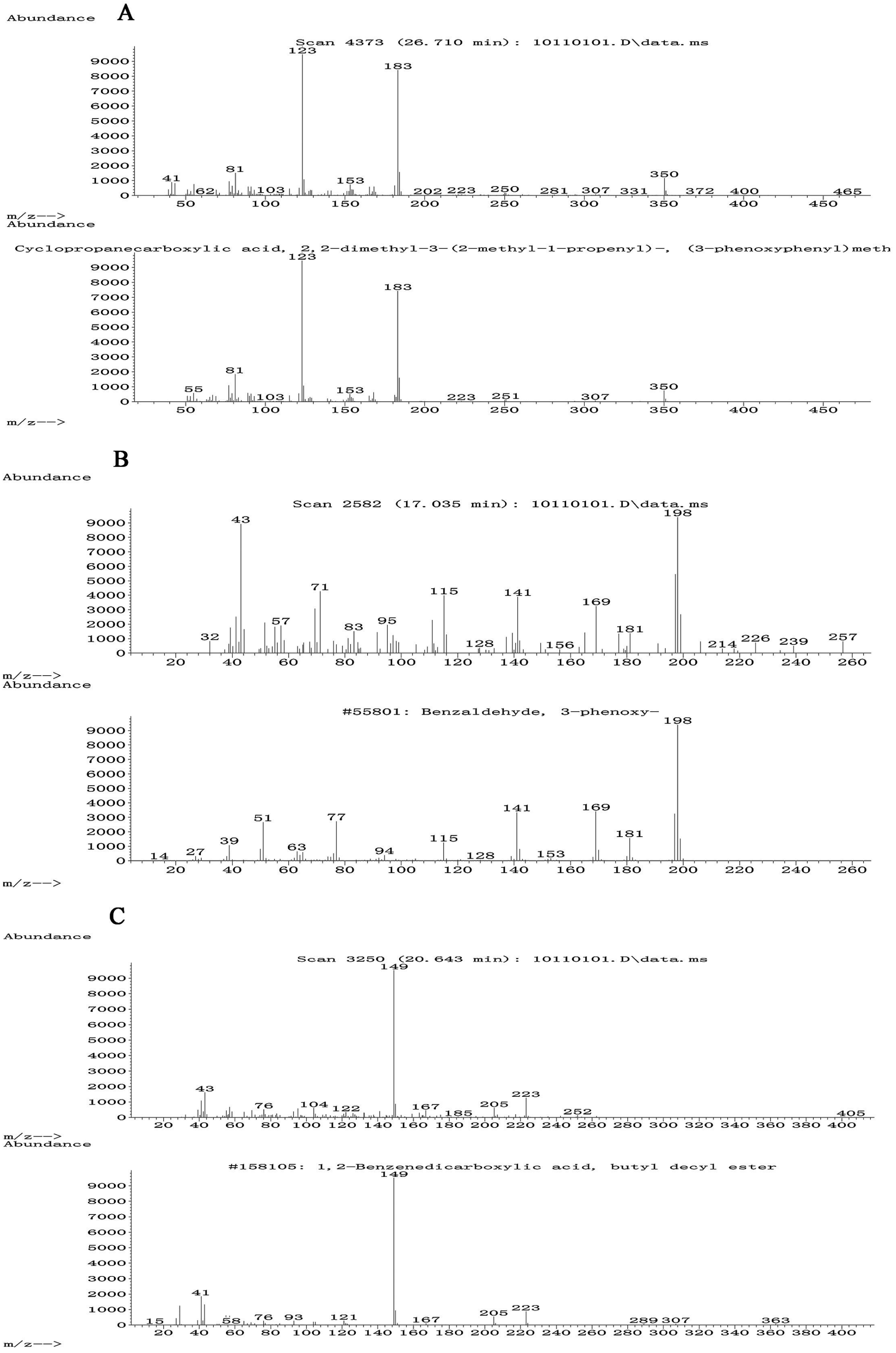

Supplementary Figure 3. The mass spectra of D-phenothrin biodegradation products reported in the National Institute of Standards and Technology (NIST, USA) library database. (A) D-phenothrin; (B) 3-phenoxybenzaldehyde; (C) 1,2-benzenedicarboxylic butyl dacyl ester.

Supplementary Tables

Supplementary Table 1 Box-Behnken experimental design matrix and the response of dependent variable for D-phenothrin degradation by strain P31

				Response (*Y*)
Run	X_1	X_2	X_3	Degradation (%)
1	25	6	0.2	85.8
2	25	8	0.2	89.1
3	35	6	0.2	80.4
4	35	8	0.2	84.5
5	30	6	0.1	90.9
6	30	6	0.3	94.0
7	30	8	0.1	89.7
8	30	8	0.3	98.6
9	25	7	0.1	88.2
10	30	7	0.1	85.6
11	25	7	0.3	93.3
12	35	7	0.3	90.4

13	30	7	0.2	100
14	30	7	0.2	100
15	30	7	0.2	100

X_1 refers to temperature (°C); X_2 refers to pH; X_3 refers to inoculum size ($g \cdot L^{-1}$).

Supplementary Table 2 Degradation of D-phenothrin by each isolated strain within 72 h of incubation

Isolated strain	Degradation (%)	Isolated strain	Degradation (%)
P01	34.2	P18	23.7
P02	13.7	P19	66.1
P03	26.4	P20	34.8
P04	12.7	P21	42.3
P05	9.5	P22	23.4
P06	72.7	P23	29.6
P07	53.4	P24	76.2
P08	23.1	P25	35.7
P09	16.9	P26	69.3
P10	25.8	P27	12.1
P11	33.2	P28	7.8
P12	27.1	P29	14.4

P13	66.4	P30	33.6
P14	81.6	P31	100
P15	11.7	P32	26.5
P16	4.6	P33	59.3
P17	12.3	P34	47.4

Supplementary Table 3 Physio-biochemical characteristics of strain P31

Characteristics	Results	Characteristics	Results
Gram staining	–	Motility	＋
Polar flagella	＋	Spore	–
Citrate utilization	＋	Anaerobic test	–
Arginine	＋	Oxidase	＋
Indole production	–	Urease production	–
Gelatin liquefaction	–	Esculin hydrolysis	–
Catalase	＋	Nitrate reduction	–

＋, tested positive; －, tested negative

Supplementary Table 4 Analysis of variance (ANOVA) for the fitted quadratic polynomial model for D-phenothrin degradation by strain P31

Source	DF	SS	MS	*F* Value	*P* Level *
X_1	1	30.0312	30.0312	29.5438	0.0029
X_2	1	14.58	14.58	14.3433	0.0013
X_3	1	59.9512	59.9512	58.9781	0.0006
X_1X_1	1	332.3544	332.3544	326.9596	0.0001
X_1X_2	1	0.16	0.16	0.1574	0.7079
X_1X_3	1	0.0225	0.0225	0.0221	0.0225
X_2X_2	1	114.2452	114.2452	112.3907	0.0001
X_2X_3	1	8.41	8.41	8.2735	0.0347
X_3X_3	1	4.7775	4.7775	4.6999	0.0824
Model	9	532.2708	59.1412	58.1812	0.0002
Error	5	5.0825	1.0165		
Total	14	537.3533			

$R^2 = 0.9905$, $adj.R^2 = 0.9735$. DF refers to degrees of freedom; SS refers to sum of sequences; MS refers to mean square. * *P* Level less than 0.05 indicates the model terms are significant.
